# Dr. Kane

**Published:** 1857-06

**Authors:** 


*Dr. Kane.*—At a recent meeting of the N. Y. Academy of Medicine, Dr. J. W. Francis stated a fact derived from the late Dr. Kane's own lips, "that his greatest weight had been 97 pounds, and that during a portion of his Arctic career, it did not exceed 93 pounds." What a physical conformation to encounter the trials and hardships through which it was his lot to pass !— *Western Lancet.*

